# *Staphylococcus aureus* small colony variants show common metabolic features in central metabolism irrespective of the underlying auxotrophism

**DOI:** 10.3389/fcimb.2014.00141

**Published:** 2014-10-21

**Authors:** André Kriegeskorte, Stephanie Grubmüller, Claudia Huber, Barbara C. Kahl, Christof von Eiff, Richard A. Proctor, Georg Peters, Wolfgang Eisenreich, Karsten Becker

**Affiliations:** ^1^Institute of Medical Microbiology, University of MünsterMünster, Germany; ^2^Lehrstuhl für Biochemie, Technische Universität MünchenGarching, Germany; ^3^Department of Microbiology and Immunology, University of Wisconsin School of Medicine and Public HealthMadison, WI, USA

**Keywords:** *S. aureus*, SCV, metabolism, TCA

## Abstract

In addition to the classical phenotype, *Staphylococcus aureus* may exhibit the small colony-variant (SCV) phenotype, which has been associated with chronic, persistent and/or relapsing infections. SCVs are characterized by common phenotypic features such as slow growth, altered susceptibility to antibiotic agents and pathogenic traits based on increased internalization and intracellular persistence. They show frequently auxotrophies mainly based on two different mechanisms: (i) deficiencies in electron transport as shown for menadione- and/or hemin-auxotrophs and (ii) thymidylate biosynthetic-defective SCVs. To get a comprehensive overview of the metabolic differences between both phenotypes, we compared sets of clinically derived menadione-, hemin- and thymidine-auxotrophic SCVs and stable site directed mutants exhibiting the SCV phenotype with their corresponding isogenic parental strains displaying the normal phenotype. Isotopologue profiling and transcriptional analysis of central genes involved in carbon metabolism, revealed large differences between both phenotypes. Labeling experiments with [U-^13^C_6_]glucose showed reduced ^13^C incorporation into aspartate and glutamate from all SCVs irrespective of the underlying auxotrophism. More specifically, these SCVs showed decreased fractions of ^13^C_2_-aspartate and glutamate; ^13^C_3_-glutamate was not detected at all in the SCVs. In comparison to the patterns in the corresponding experiment with the classical *S. aureus* phenotype, this indicated a reduced carbon flux via the citric acid cycle in all SCV phenotypes. Indeed, the aconitase-encoding gene (*acnA*) was found down-regulated in all SCV phenotypes under study. In conclusion, all SCV phenotypes including clinical isolates and site-directed mutants displaying the SCV phenotype were characterized by down-regulation of citric acid cycle activity. The common metabolic features in central carbon metabolism found in all SCVs may explain similar characteristics of the *S. aureus* SCVs irrespective of their auxotrophism as well as the specific genetic and/or regulatory backgrounds.

## Introduction

*Staphylococcus aureus* (*S. aureus*) has been recognized as one of the most important human pathogens world-wide causing a wide range of mild to serious infections within and outside the hospital aggravated by the dissemination of different methicillin-resistant *S. aureus* (MRSA) lineages (Lowy, 1998; David and Daum, 2010). Besides its capability to cause acute infections, *S. aureus* can cause chronic courses of infection despite adequate antimicrobial therapy that are often associated with a defined *S. aureus* phenotype, designated as small-colony variants (SCVs) (Proctor et al., 2006). SCVs represent a sub-population with distinct phenotypic and pathogenic traits adapted to an intracellular lifestyle (von Eiff et al., 2001, 2006; Sachse et al., 2010; Tuchscherr et al., 2010). As main feature, they show frequently auxotrophies (auxotrophism) for menadione, hemin and/or thymidine, however, strains without any detectable auxotrophy or with other auxotrophies including those for CO_2_ and thiamin have been described (Thomas, 1955; von Eiff et al., 1997; Kahl et al., 2003; Chatterjee et al., 2008; Lannergård et al., 2008; Gómez-González et al., 2010). The best-investigated and most prevalent SCV phenotypes, the menadione and/or hemin autotrophic SCVs as well as thymidine autotrophic SCVs, are characterized by deficiencies in the electron transport and in the thymidylate biosynthetic pathway, respectively (von Eiff et al., 1997; Chatterjee et al., 2008). It has been shown for hemin and menadione auxotrophic SCVs, based on mutations in *hemB* and *menD* (von Eiff et al., 1997; Kohler et al., 2003, 2008), that genes involved in the central metabolic processes were affected. Transcriptomic and proteomic approaches revealed considerable differences between the wild type and SCV phenotypes especially in the fermentative pathways (Kohler et al., 2003, 2008; Seggewiss et al., 2006). However, because clinically derived SCVs tend to revert quickly back into the wild type phenotype, most of SCV studies were performed with genetically defined, stable mutants. A recent proteomic study comparing a clinically derived SCV with a corresponding *hemB* mutant SCV and a gentamicin-induced SCV revealed common, but also distinct features between naturally occurring and genetically generated SCVs apart from changes triggered by the mutational inactivation of the electron transport chain (Kriegeskorte et al., 2011). Nevertheless, the complex metabolic and physiological changes along with the SCV phenotype are still not fully understood and more multifaceted than revealed from studies with genetically defined mutants displaying the SCV phenotype.

The aim of this study was to get additional insights into the metabolic properties of *S. aureus* SCVs as compared to their corresponding isogenic normal phenotype. For this purpose, we investigated a comprehensive set of SCVs including both clinically derived strains and stable site directed mutants by ^13^C-isotopologue profiling and transcriptional analysis.

## Materials and methods

### Bacterial strains and culture conditions

Clinical *S. aureus* wild types and SCVs were recovered in parallel from patients with chronic infections (e.g., osteomyelitis and cystic fibrosis). Clonality was verified by *Sma*I macrorestriction analyses by pulsed-field gel electrophoresis (PFGE). Strains used in this study were summarized in Table 1. The *S. aureus* isolates were grown on Columbia sheep blood agar and tryptic soy agar at 37°C for 24–48 h. Liquid cultures were grown aerobically in 50 ml tryptic soy broth (TSB) in 500 ml flasks at 37°C and 160 rpm. For labeling experiments (isotopolog profiling) TSB without dextrose (Bacto Tryptic Soy without dextrose, BD, New Jersey, USA) including 17 g of pancreatic digest of casein, 3 g of enzymatic digest of sojabean meal, 5 g of sodium chloride and 2.5 g of dipotassium phosphate was used. The medium was supplemented with 2.5 g of [U-^13^C_6_]glucose.

**Table 1:** **Bacterial strains used in this study**.

**Strain designation in this study**	**Description**	**Source**
**CLINICALLY DERIVED STRAIN PAIRS**		
I^WT^	Clinical wild-type (strain 1549I)	This study
I^SCV^	Clinical SCV; heme auxotroph (strain 1549III)	This study
III^WT^	Clinical wild-type (strain F2418II)	Chatterjee et al., 2008
III^SCV^	Clinical SCV; thymidine auxotroph (strain F2418)	Chatterjee et al., 2008
V^WT^	Clinical wild-type (strain 22616/1)	Lannergård et al., 2008
V^SCV^	Clinical SCV; menadione auxotroph (strain 22616/3)	Lannergård et al., 2008
WT^3878^	Clinical wild-type (strain 3878I)	Kriegeskorte et al., 2011
cSCV^3878^	Clinical SCV (strain3878III)	Kriegeskorte et al., 2011
**STABLE *IN VITRO* GENERATED MUTANTS**		
II^WT^	Wild type (strain 8325-4)	O'Neill, 2010
II^SCV^	*ΔhemB* SCV (strain 8325-4)	This study
IV^WT^	Wild type (strain SH1000)	O'Neill, 2010
IV^SCV^	*ΔthyA* SCV (strain SH1000)	Kriegeskorte et al., 2012
VI^WT^	Wild type (strain 6850)	Fraunholz et al., 2013
VI^SCV^	Menadione auxotroph SCV (strain JB1; generated from strain 6850)	Balwit et al., 1994
*ΔhemB*^3878^	Site directed *hemB* mutant SCV (generated from wild-type strain 3878I)	Kriegeskorte et al., 2011
KM^3878^	Complementation of *ΔhemB*^3878^ with pCX19 *hemB*	Kriegeskorte et al., 2011
REV^3878^	Spontaneous revertant strain of cSCV^3878^	Kriegeskorte et al., 2011
G^3878^	Gentamicin -induced SCV of WT^3878^ (menadione auxotroph)	Kriegeskorte et al., 2011

### Cell isolation and growth curve analysis

For isotopolog profiling, 50 ml cultures were inoculated to an optical density of 0.05 (578 nm) from overnight cultures. Cells were harvested after 540 min by centrifugation (10 min, 5000 × g, 4°C) and washed three times with 10 ml PBS. Pellets were stored at −80°C. Cells were resuspended in 10 ml PBS and autoclaved (20 min, 121°C). For the growth curve analysis, cultures were grown in 50 ml TSB in 500 ml flasks at 37°C on a rotary shaker at 160 rpm. The optical density was measured every hour at 578 nm using Ultraspec 1100 pro spectrophotometer (Amersham Biosciences, Freiburg, Germany).

### Construction of a Δ*hemB*-mutant in *S. aureus* 8325-4

The *hemB* knockout mutant of *S. aureus* 8325-4 was constructed by allelic replacement of the *hemB* gene with *ermB* cassette (mediating erythromycin resistance) using the vector pCE8 as described before (von Eiff et al., 1997). The mutant was verified by restriction analysis and sequencing.

### Isotopologue profiling

Bacterial cells (approximately 5 mg) were suspended in 0.5 ml of 6 M hydrochloric acid and incubated at 105°C for 24 h. The amino acids were purified on a Dowex 50W×8 column (washing 2 × 750 μl H_2_0; developing 1 ml 2 M ammonium hydroxide). The eluate was dried under a steam of nitrogen and resuspended in 50 μl dry acetonitrile. 50 μl of N-(tert-butyldimethylsilyl)-N-methyl-trifluoroacetamide containing 1 % of tert-butyldimethylsilylchlorid were added and the mixture was incubated at 70°C for 30 min. The tert-butyl-dimethylsilyl derivatives of amino acids were then used for gas chromatography–mass spectrometry (GC/MS) and isotopolog analysis as described elsewhere (Eylert et al., 2008).

### Semi-quantitative RT-PCR

Total RNA was extracted from bacteria grown in TSB medium to late exponential growth phase using QuantiTect reverse transcription kit (QIAGEN) according to the manufacturer's recommendations. PCR reaction were performed with the CFX96 system (Bio-Rad Laboratories, München, Germany) under the following conditions: 95°C for 15 min, 50 cycles (95°C for 10 s, 10 s at 55°C for 10 s, 72°C for 30 s) using the EvaGreen Kit (Segentic, Borken, Germany). Three independent biological replicates were tested in duplicate. N-fold expression values relative to the house-keeping genes *gyrB, gmk* and *aroE* (normalized by the geometric mean of the relative quantities of all three reference genes) and for each strain set normalized to expression of the wild type isolate were calculated using CFX Manager v3.1 (Bio-Rad).

## Results and discussion

*S. aureus* SCVs show many common features, such as slow growth, reduced pigmentation and changed expression of virulence determinants, independent of their underlying auxotrophic phenotype, molecular mechanism of SCV phenotype generation or genetic strain background. A similar phenotypic appearance may reflect analogous metabolic properties or a similar metabolic status. To investigate the metabolic differences (i) between *S. aureus* normal and SCV phenotypes and (ii) between different kinds of SCVs by isotopolog profiling, respective isogenic strains sets were analyzed (Table 1) and regulatory differences in central metabolic and virulence related genes were determined.

**Figure 1:**
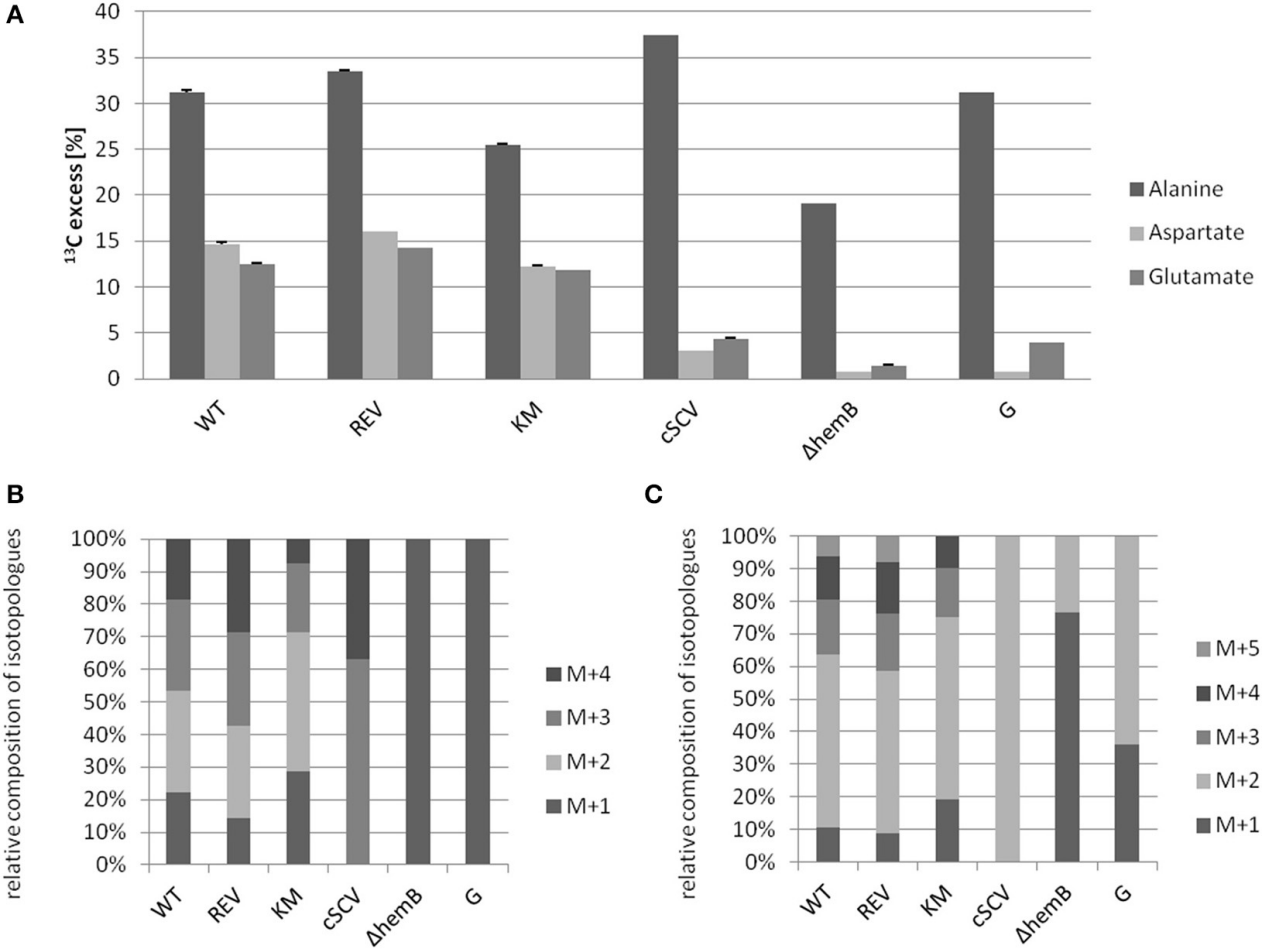
**^13^C-Excess in alanine, aspartate and glutamate from *S. aureus* strains grown with [U-^13^C_6_]glucose (A)**. Error bars indicate the standard deviation of three technical replicates. WT (clinically isolated normal phenotype), REV (spontanous revertant of the clinical SCV) and KM (complemented Δ*hemB* mutant with an intact *hemB* gene in trans) display an *S. aureus* wild-type phenotype. cSCV (clinically isolated SCV phenotype), Δ*hemB* (site-directed *hemB* mutant of WT) and G (gentamicin induced SCV phenotype) display a *S. aureus* SCV phenotype. Relative contribution of ^13^C-isotopologs carrying one to five ^13^C-atoms (M+1–M+5) in aspartate **(B)** and glutamate **(C)**. The group of M+X comprises all isotopologs of aspartate and glutamate with only X labeled carbon units irrespective of the location within the molecule. Isotopologue patterns of amino acids with <1% ^13^C-excess are not shown due to the high standard deviations.

Using a *S. aureus* strain “sextet,” consisting of three wild type isolates and three isolates displaying different SCV phenotypes including a clinically derived SCV, a site-directed *hemB* mutant and a gentamicin induced SCV, we identified significant phenotypic specific differences in the labeling patterns of amino acids (Figures 1A–C) (Kriegeskorte et al., 2011). In experiments with 2.5 g/l [U-^13^C_6_]glucose as a supplement to the TSB medium, all isolates, independent of their phenotype, showed high ^13^C incorporation of about 20–40% into alanin (Figures 1A, **7A**), reflecting a high glycolytic activity in both phenotypes. As expected, under *in vitro* nutrient rich conditions, glucose served as the major energy source for growth of *S. aureus*. Nevertheless, the fraction of unlabeled amino acids in the experiment with [U-^13^C_6_]glucose reflected the pronounced capacity of *S. aureus* to uptake and to use external (unlabeled) amino acids or peptides from the medium. In comparison to alanine, more pronounced differences were noticed in the labeling patterns of glutamate and aspartate (Figures 1B,C). While all normal phenotypes showed a ^13^C-excess between 11.8 and 16.0% in aspartate and glutamate, all SCV phenotypes were characterized by a substantial reduced ^13^C-excess between 0.7 and 4.4% (Figure 1A). As glutamate and aspartate are directly linked to the citric acid cycle intermediates α-ketoglutarate and oxalacetate, respectively, via transamination, the reduced ^13^C-label of these amino acids could indicate a reduced citric acid cycle activity in all SCV phenotypes (**Figure 5**). The comparison of the averaged ^13^C-excess values between the three normal and the three SCV phenotypes revealed no significant differences in the ^13^C-excess of alanine, but a significant reduction of the ^13^C-excess of aspartate and glutamate in the SCV phenotypes (**Figure 7A**). Moreover, the isotopolog distributions in aspartate and glutamate from the SCVs were clearly different from the corresponding patterns in aspartate and glutamate from the wild-type phenotype and the revertant or complemented strains (Figures 1B,C). Whereas the later group was characterized by multiple ^13^C-isotopologs also comprising **three** and more ^13^C-atoms, the amino acids from the SCVs displayed higher fractions of ^13^C_1_-isotopologs. Again, this could reflect that the carbon flux via the citrate cycle producing oxaloacetate/Asp and α-ketoglutarate/Glu carrying multiple ^13^C-atoms in the experiments with [U-^13^C_6_]glucose is substantially reduced in the *hemB* mutant and the gentamicin induced SCVs. This hypothesis is in line with earlier conclusions made on the basis of proteome and transcriptome studies (Kohler et al., 2003, 2008; Seggewiss et al., 2006).

**Figure 2:**
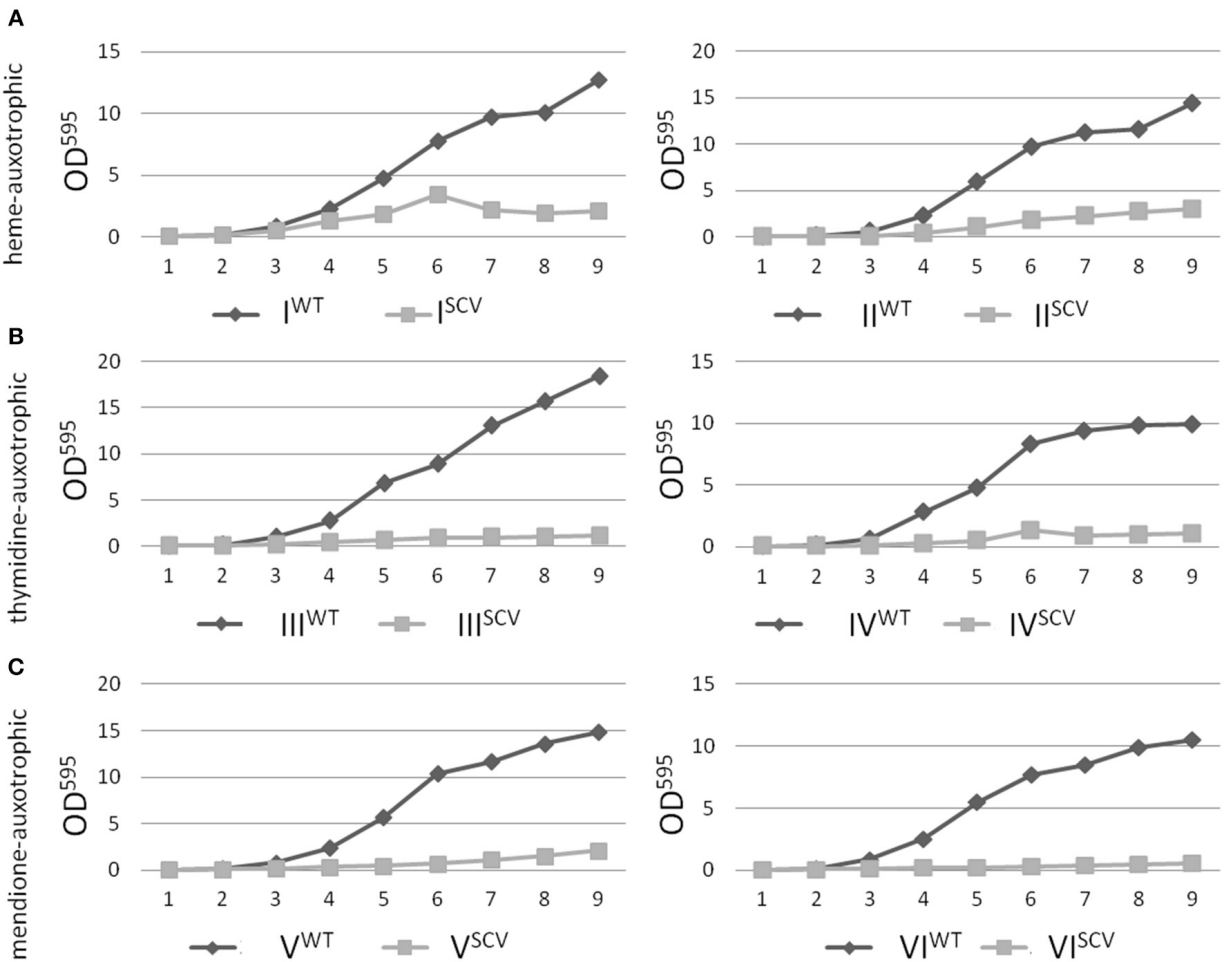
**Growth curve analysis of *S. aureus* isolates with normal and SCV phenotype**. Heme-auxotrophic **(A)**, thymidine-auxotrophic **(B)** and menadione-auxotrophic **(C)**. Strain pairs I, III, and V represent clinical isolates consisting of a normal phenotype (WT) and a SCV phenotype (SCV). Strain pairs II, IV, and VI consist of a normal phenotype and an *in vitro* generated SCV phenotype. The optical density of the cultures was measured at 595 nm and values were plotted against the time.

**Figure 3:**
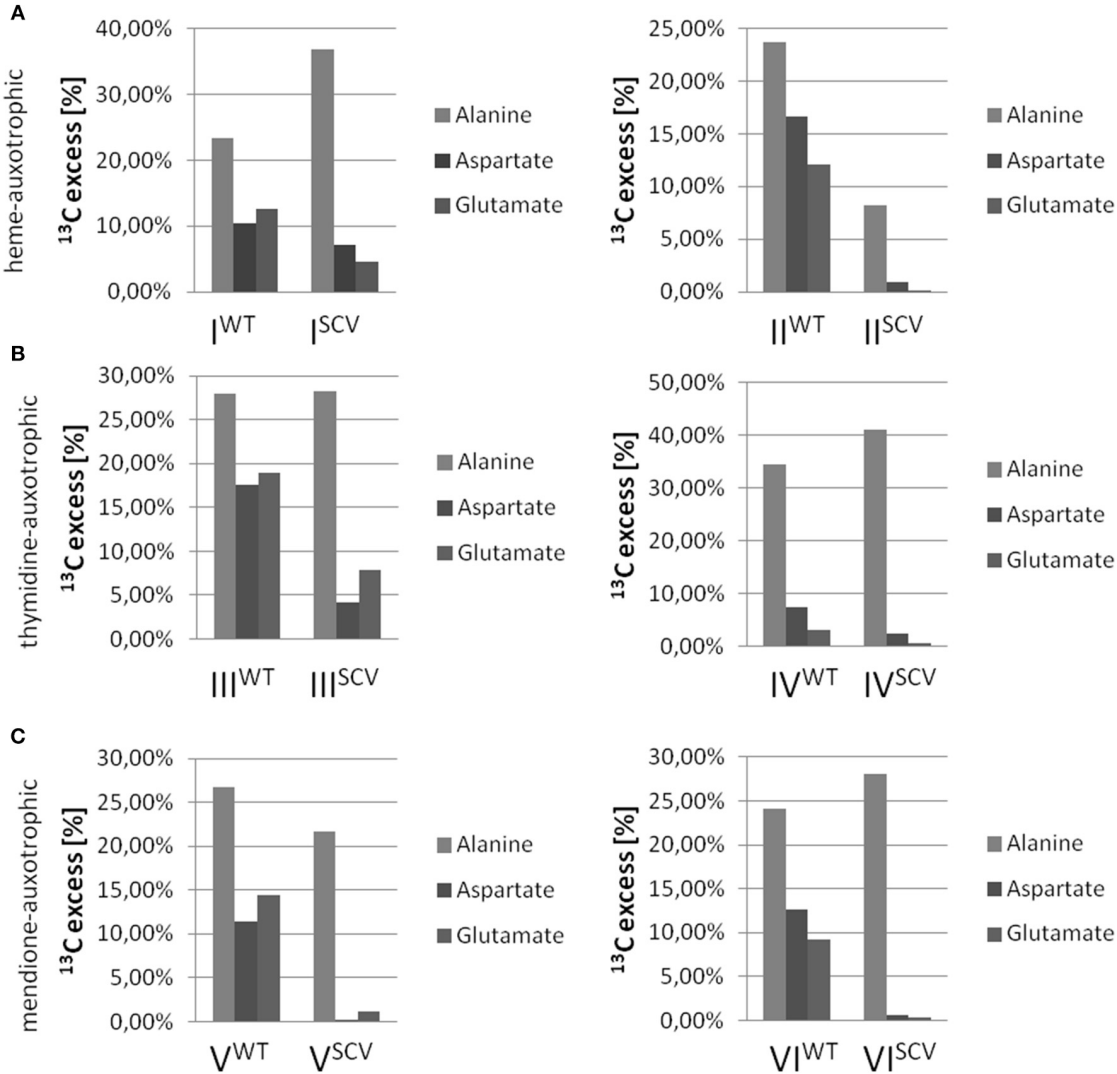
**^13^C-Excess in alanine, aspartate and glutamate from *S. aureus* strains grown with [U-^13^C_6_]glucose**. Columns represent the mean values of three technical replicates; heme-auxotrophic **(A)**, thymidine-auxotrophic **(B)** and menadione-auxotrophic **(C)**. Strain pairs I, III, and V represent clinical isolates consisting of a normal phenotype (WT) and a SCV phenotype (SCV). Strain pairs II, IV, and VI consist of a normal phenotype and an *in vitro* generated SCV phenotype.

**Figure 4:**
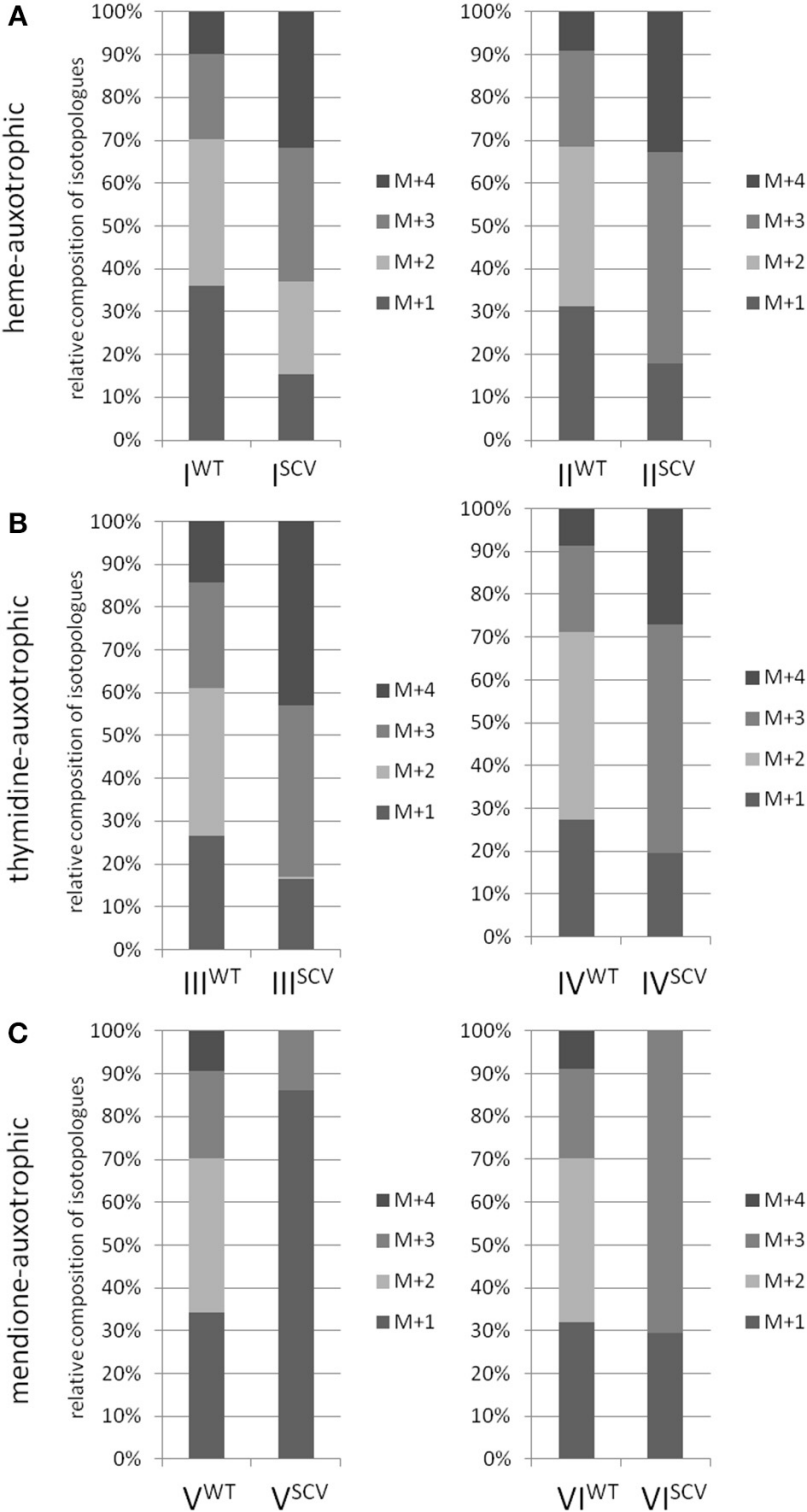
**Isotopologue composition of aspartate**. Comparison between normal and SCV phenotypes of *S. aureus* displaying different auxotrophisms. Heme-auxotrophic **(A)**, thymidine-auxotrophic **(B)** and mendione-auxotrophic **(C)**. Strain pairs I, III, and V represent clinical isolates. Strain pairs II, IV, and VI consist of a normal phenotype and an *in vitro* generated SCV phenotype. Patterned columns represent the mean values of three technical replicates. The relative contributions of each ^13^C-isotopolog (M+1–M+4) with regard to the overall enrichment were shown.

**Figure 5:**
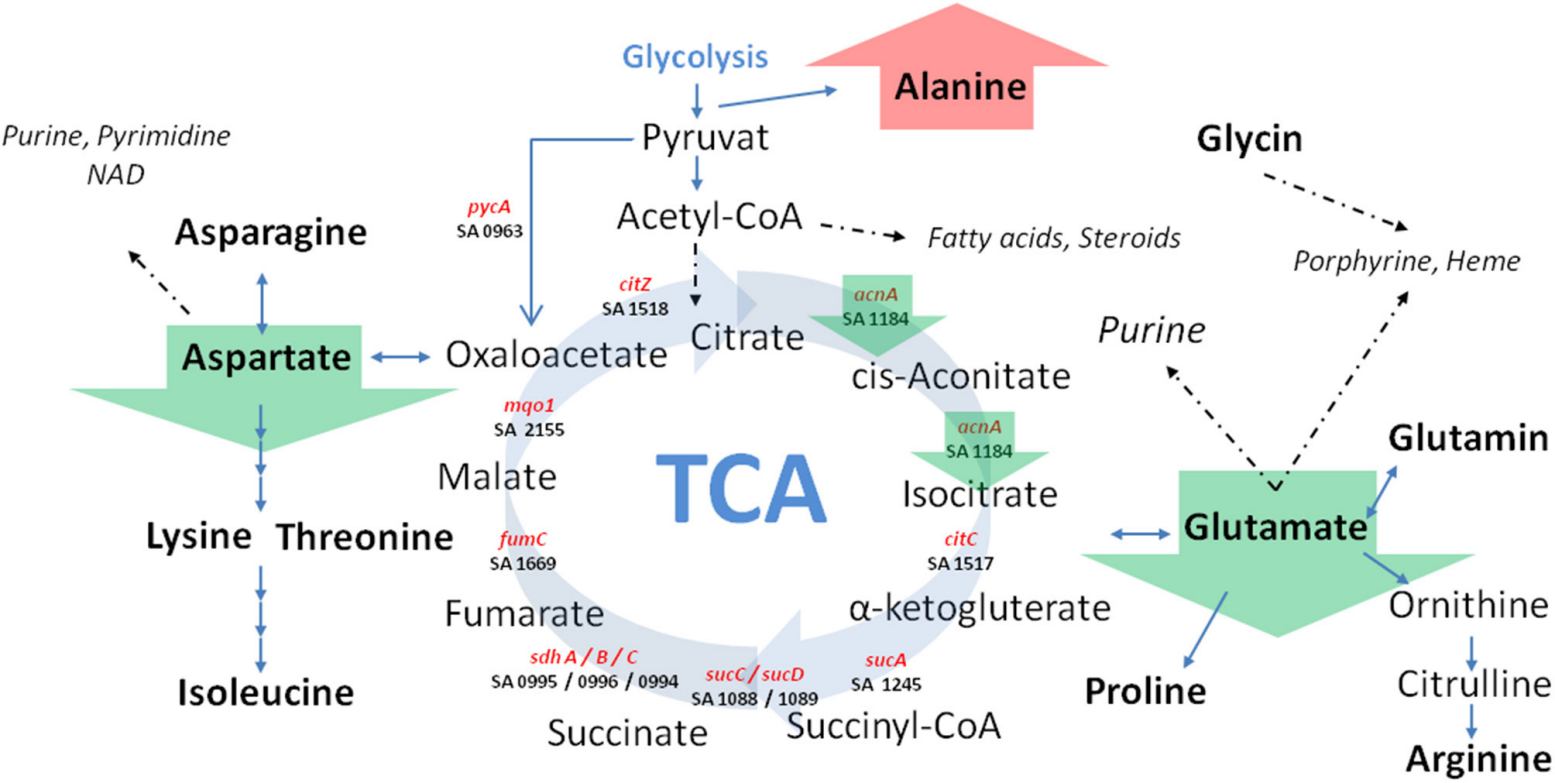
**Schematic view of central carbon metabolism in *S. aureus***. Green arrows marked SCV specific differences irrespective of the underlying auxotrophism compared to their corresponding normal phenotypes. TCA: tricarboxylic acid cycle; Involved genes (marked in red) and corresponding KEGG gene identifier were shown.

Interestingly, the clinical SCV showed similar patterns of the ^13^C-excess in aspartate and glutamate, in comparison to the profiles from the site-directed *hemB* mutant and to the gentamicin induced SCV, respectively (Figure 1A). However, the relative isotopolog distributions differed between the SCV phenotypes (Figures 1B,C). While in the *hemB* mutant and in the gentamicin induced SCV, the M+1 species were dominant, the 13C_2_-species were more abundant indicating that single runs, but no multiple runs, via the citric acid cycle were still operative in the clinically derived SCV. To investigate whether reduced carbon flux via the citrate cycle is a general feature of *S. aureus* SCVs that also includes the major auxotrophic phenotypes (hemin, menadione, and thymidine), we analyzed a comprehensive set of six isogenic strain sets, each comprising the normal wild type and different SCV phenotypes including clinical derived SCVs and genetically defined mutants displaying the SCV phenotype. The growth properties of the strain pairs are shown in Figures 2A–C, **7B**. All SCVs showed a pronounced growth defect compared to their normal phenotypes, irrespective of the underlying auxotrophism and reached considerably lower optical densities under aerobic conditions. Again, ^13^C incorporation into alanine was highly efficient in all phenotypes and strains and no significant difference between the normal and the SCV phenotypes could be observed (Figures 3A–C, **7B**). However, irrespective of the underlying auxotrophism, all SCV phenotypes again showed reduced ^13^C-incorporation into aspartate and glutamate, revealing the assumed reduced activity of carbon flux via the citric acid cycle (Figures 3A–C, **7B**). This conclusion was verified by the isotopolog distribution as shown in Figures 4A–C. While all of the normal phenotypes showed in aspartate high fractions of the M+2 species, all SCVs were devoid of this species. In contrast, the SCV phenotypes showed increased relative fractions of M+1 and M+3 species indicating the reduced flux via the citric acid cycle with ^13^C_2_-isotopologs, but higher contributions of oxaloacetate/Asp formation by anaplerotic reactions [i.e., giving rise to the ^13^C_3_-isotopologs by carboxylation of [U-^13^C_3_]pyruvate or PEP in the SCVs (Figure 5)].

**Figure 6:**
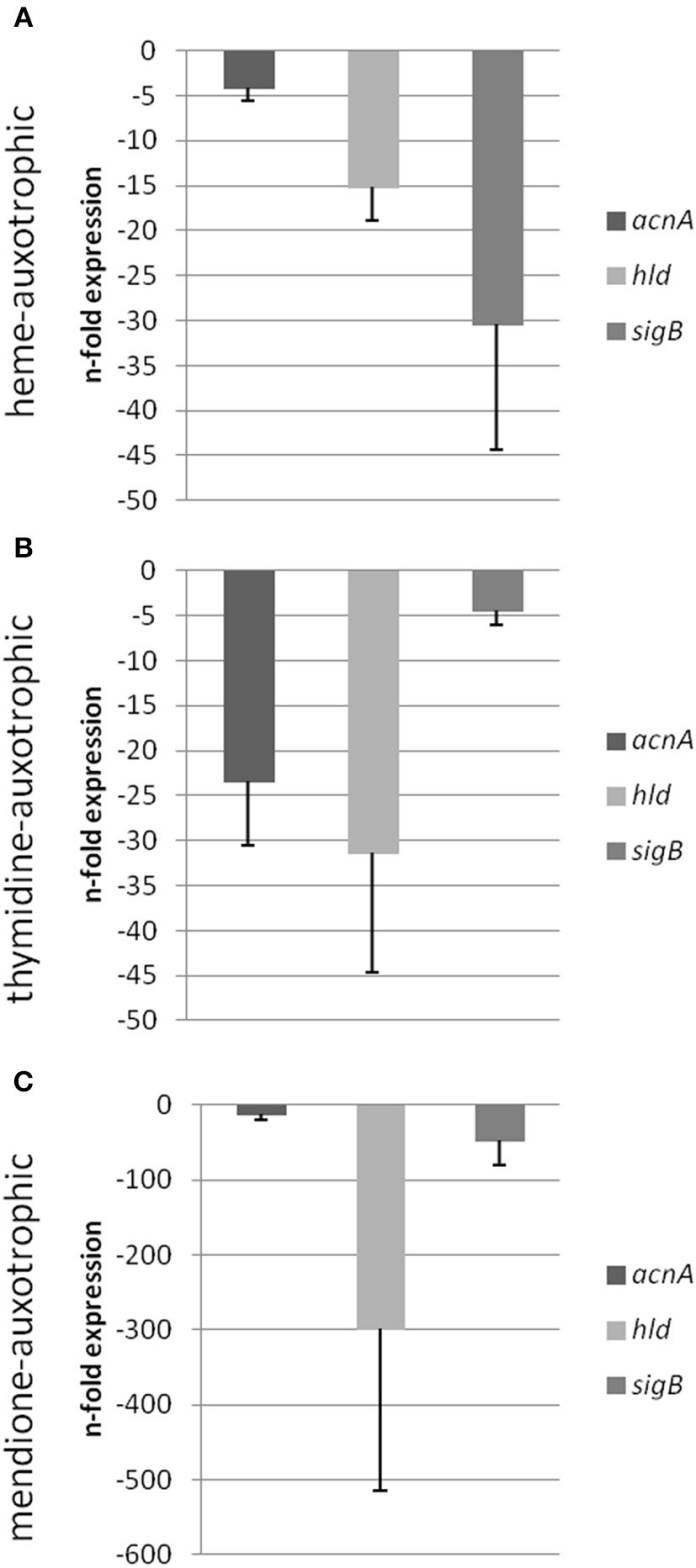
**Semi-quantitative rt-PCR**. Quantification of the expression of *acnA, hld* and *sigB* of different *S. aureus* strains displaying the SCV phenotype compared to its respective isogenic wild-type isolate. Expression data is normalized using three internal control genes (*gmk, aroE*, and *gyrB*) and displayed relative to the corresponding wild-type isolates (I^WT^, III^WT^, and V^WT^ were set as 1). Isogenic strain pair I^WT^ and I^SCV^ [heme auxotrophic **(A)**], isogenic strain pair III^WT^ and III^SCV^ [thymidine auxotrophic **(B)**] and isogenic strain pair V^WT^ and V^SCV^ [menadione auxotrophic **(C)**] were shown.

**Figure 7:**
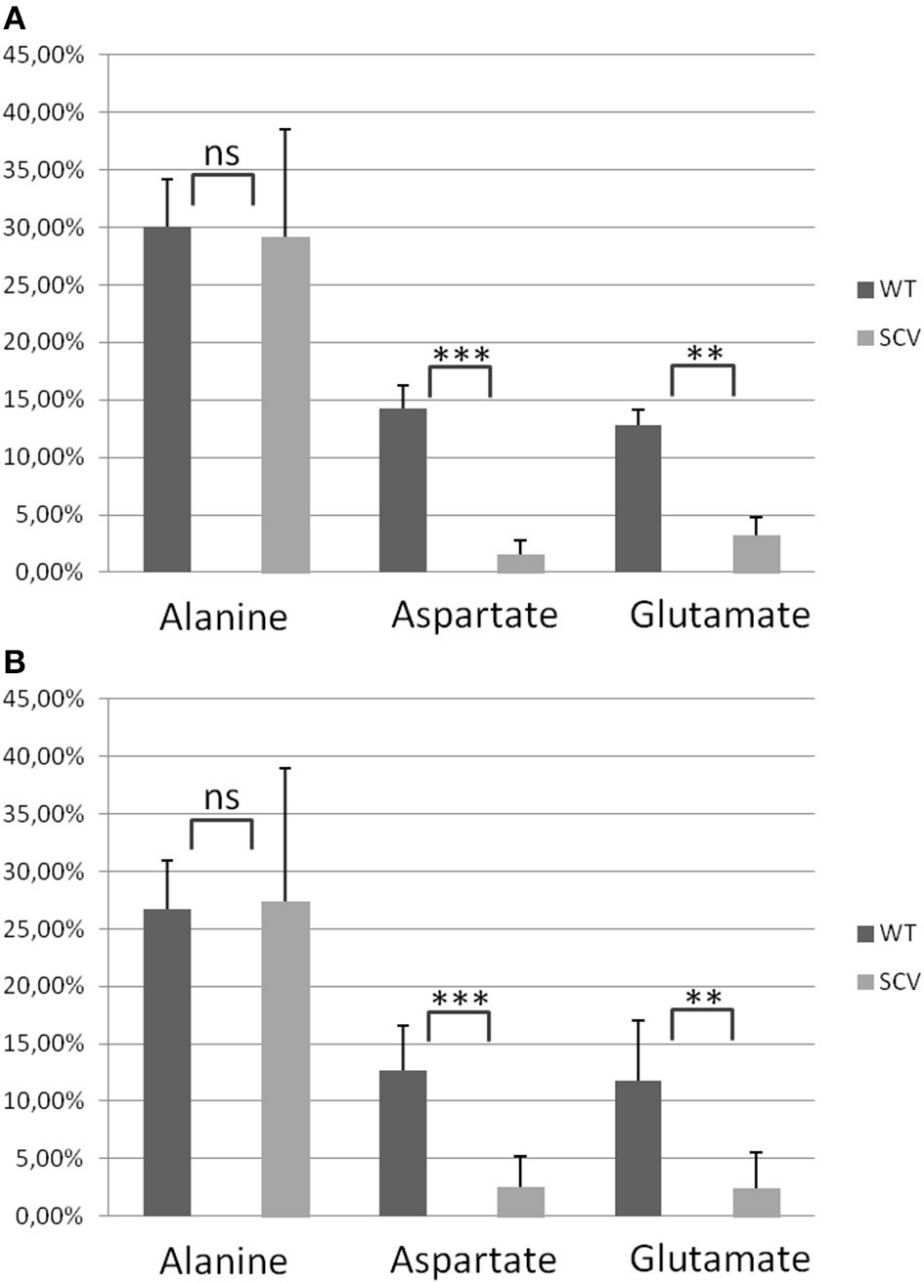
**Averaged ^13^C-Excess in alanine, aspartate and glutamate from *S. aureus* normal and SCV phenotypes. (A)** Columns represent the averaged values ± standard deviation (SD) of the normal phenotypes (WT) including *S. aureus* 3878 WT, REV and KM and the SCV phenotypes (SCV) including *S. aureus* cSCV, Δ*hemB* and G. **(B)** Columns represent the averaged values ± standard deviation (SD) of the normal phenotypes (WT) including *S. aureus* I-VI^WT^ and the SCV phenotypes (SCV) including *S. aureus* I-VI^SCV^. Statistical significance was assessed in pairwise comparison between WT and SCV for each amino acid with the two-tailed Student's *t*-test. ^**^P < 0.01, ^***^P < 0.001.

Previously, we could show that the expression of *acnA* (aconitase) which catalyzes the first step of the TCA cycle (Figure 5), was reduced in a clinical SCV as well as in a site-directed *hemB* mutant (Seggewiss et al., 2006; Al Laham et al., 2007; Kriegeskorte et al., 2011). With the recent isotopolog data, down-regulation of *acnA* resulting in reduced carbon flux via the citric acid cycle seems to be a common feature of the SCV metabolism irrespective of the underlying molecular mechanism leading to this phenotype. Not surprisingly, all of the investigated clinical SCVs comprising heme, menadione and thymidine auxotrophic phenotypes, respectively, displayed a transcriptional down-regulation of *acnA* compared to their corresponding normal phenotype (Figures 6A–C, 7A,B). This is in line with previous studies on transcriptomic or proteomic level (Kohler et al., 2003; Seggewiss et al., 2006). Along with the decreased metabolic activity, all SCVs showed markedly reduced expression of the major virulence regulators *hld* and *sigB*. Hld is the effector molecule (a regulatory RNA) of the agr system which regulates virulence determinants in *S. aureus* such as the major toxins *hla* (α-hemolysin) and *hlb* (β-hemolysin). Corresponding to the reduced expression of *hld*, the investigated SCV phenotypes showed a clearly reduced hemolytic activity on Columbia blood agar plates (data not shown).

In conclusion, all SCV phenotypes irrespective of their auxotrophism and genetic background revealed down-regulation of citric cycle activity as shown by the reduced ^13^C-incorporation into aspartate and glutamate with modified isotopolog profiles, as well as by down-regulation of *acnA* on the transcriptional level. A reduced metabolic status of all kinds of SCVs may explain the concordant major characteristics of the *S. aureus* SCV phenotypes regardless of the mechanism of their formation.

### Conflict of interest statement

The Guest Associate Editor Thomas Dandekar declares that, despite having collaborated with authors Claudia Huber (Eisenreich group) and Wolfgang Eisenreich (Co-Topic Editor of this issue), the review process was handled objectively and no conflict of interest exists. The authors declare that the research was conducted in the absence of any commercial or financial relationships that could be construed as a potential conflict of interest.
